# Molecular Imprinting of Phosphate Moieties into the Silica Matrix as a Novel Phosphorus Rechargeable System for Copper Ions Adsorption

**DOI:** 10.3390/polym18141759

**Published:** 2026-07-18

**Authors:** José A. Gutiérrez-Ortega, Jessica Badillo-Camacho, Rene G. Moran-Salazar, Sergio Gómez-Salazar, Ilya G. Shenderovich, Yenni G. Velázquez-Galván, Ricardo Manríquez-González

**Affiliations:** 1 Department of Wood, Cellulose and Paper, University of Guadalajara-CUCEI, Km 15.5 of Carretera Guadalajara-Nogales, Zapopan 45020, Mexico; joseantonio.gutierrez@academicos.udg.mx; 2 Department of Chemistry, Universidad de Guadalajara-CUCEI, Blvd. Marcelino García Barragán #1421, Guadalajara 44430, Mexico; rene.moran@academicos.udg.mx; 3 Department of Chemical Engineering, Blvd. Marcelino García Barragán # 1421, Guadalajara 44430, Mexico; sergio.gomez@cucei.udg.mx; 4 Faculty of Chemistry and Pharmacy, University of Regensburg, Universitaetstrasse 31, 93053 Regensburg, Germany; 5 Department of Mathematics and Physics, Instituto Tecnológico y de Estudios Superiores de Occidente (ITESO), Periférico Sur Manuel Gómez Morín 8585, Tlaquepaque 45604, Mexico; yenni.velazquez@iteso.mx

**Keywords:** silica gel, molecular imprinting, phosphate–copper interaction, adsorbent, rechargeable cavities

## Abstract

Silica gel polymer material with imprinted phosphate cavities was successfully obtained using one-pot sol–gel reaction. Differences in the textural properties concerning the reduction in specific area and pore size between functionalized and pristine silica gel demonstrated the presence of the phosphate moieties in the cavities. The chemical and structural characterization of the functionalized material before and after copper adsorption was performed by Fourier-transform infrared spectroscopy (FTIR) and solid-state ^29^Si and ^31^P nuclear magnetic resonance (NMR) spectroscopy. All these measures proposed a phosphate non-covalently bound in the cavities of the silica gel and stabilized by silanol groups on the surface of the matrix. The phosphate–copper complex is removed after the metal desorption process, and the free cavities in the silica matrix can be replenished with phosphoric acid without affecting its adsorption capacity. The entire process of phosphate incorporation, copper adsorption, and metal-ligand desorption was repeated in three cycles, showing a similar metal adsorption capacity. Energy-dispersive X-ray spectroscopy (SEM-EDX) experiments were performed to monitor the presence and proportion of phosphorus and copper at each step of the phosphate loading and copper adsorption processes. These results demonstrate the feasibility of synthesizing a rechargeable polymer material with functional molded cavities with phosphate groups capable of adsorbing copper ions. Finally, this investigation represents the first approach to new materials with a rechargeable ligand system for the adsorption of heavy metals.

## 1. Introduction

The increasing contamination of aquatic environments with heavy metal ions has raised serious environmental and public health concerns. Among these pollutants, copper ions (Cu(II)) are particularly critical due to their widespread industrial use and toxicity at elevated concentrations in water streams [1]. In response, the development of efficient remediation materials based on adsorption processes has attracted significant attention [2,3,4,5,6].

Within this context, hybrid silica-based adsorbents synthesized via the sol–gel method have emerged as promising candidates owing to their tunable porosity, high surface area, and the ability to incorporate functional groups tailored for specific metal ion interactions [7,8,9]. Functionalization with phosphorus-based ligands, such as phosphonates and phosphates, as well as amino acid moieties, has shown effective coordination with various heavy metal cations, including Pb(II), As(V), and Cr(III) [10,11,12,13,14,15]. Despite these advances, many studies prioritize adsorption performance without investigating the stability or recyclability of the functional groups involved. Particularly when non-covalent interactions dominate the binding process.

Phosphate moieties offer an attractive alternative to covalently grafted ligands due to their ability to form stable ionic interactions with metal cations, while also being potentially desorbable and reloadable under controlled chemical conditions [16,17]. In this regard, new approaches have been proposed for the uptake or association of metal ions such as calcium in silica cavities, where the phosphate functional groups are not covalently grafted onto the silica surface, but are stabilized by silanols [16,18]. Stabilization of these phosphate groups within the silica matrix, particularly through hydrogen bonding with silanols on the surface, enables the self-design of cavities in mesoporous materials that mimic the principles of molecular imprinting during the sol–gel process. However, these studies did not report information on the reusability or the ligand reloading system of these materials. Therefore, the ligand reloading strategy could lead to reusable adsorbents capable of undergoing repeated cycles of metal ion uptake, ligand desorption, and functional replacement with profitable ligands. However, before evaluating the adsorption efficiency and recyclability of these new materials for environmental applications, an initial assessment stage is necessary to understand their viability as an adsorbent.

In this study, we report the synthesis of a phosphate-functionalized silica polymer material (SG-P) via a one-pot sol–gel process. Tetraethyl orthosilicate (TEOS) was used as the silica matrix precursor, and triethyl phosphate (TEPO) was used as the phosphorus source and the cavity templates by molecular imprinting. The resulting polymer material contains phosphate moieties non-covalently retained within the silica network. The effectiveness of the Cu(II) adsorption capacity of the material from aqueous solutions, as well as the possible loss of phosphate groups in the desorption process, is followed by SEM-EDX. Phosphoric acid was employed as a ligand reloading strategy to replenish the phosphate functionalities and restore the material’s adsorption capability. This approach establishes a novel framework for a profitable and recyclable polymer adsorbent system.

To evaluate the structure–function relationship and regeneration potential of the material, a combination of different analytical techniques of Brunauer–Emmett–Teller (BET) surface analysis, solid-state ^31^P and ^29^Si nuclear magnetic resonance (NMR) spectroscopy, energy-dispersive X-ray spectroscopy (SEM–EDX) elemental analysis, and surface acidity by potentiometric titrations was employed. The main goal is to understand the mechanism of the noncovalent ligand performance and the effectiveness of the recharging process for the heavy metal uptake. To our knowledge, no similar system on functionalized materials has been described in the literature. Therefore, these findings offer new insights into the design of reusable functionalized silica adsorbents with reloadable active sites for use in future sustainable water treatment technologies.

## 2. Materials and Methods

### 2.1. Materials

Silica gel matrix precursor tetraethyl orthosilicate (TEOS) ≥ 99% and triethyl phosphate (TEPO) as functionalizing and imprinting agent, and triethylamine (TEA) ≥ 99% were purchased from Sigma Aldrich (Toluca, Mexico). Absolute ethanol, acetone ≥ 99%, hydrochloric acid 37 wt%, nitric acid ≥ 99%, phosphoric acid ≥ 99%, and sodium chloride ≥ 99% were supplied by Fermont (Monterrey, Mexico). All reagents were used without further purification.

### 2.2. Synthesis of Adsorbent SG-P

**Figure 1:**
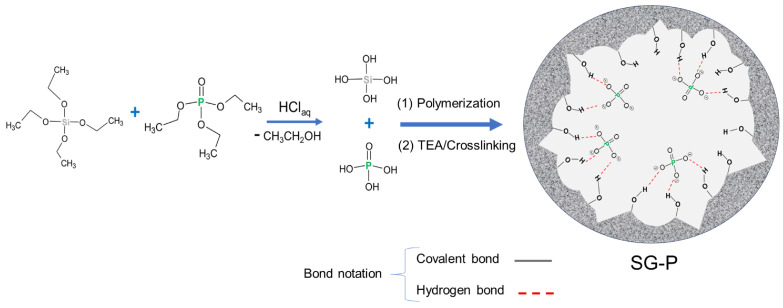
Synthesis of the silica gel with phosphates (SG-P) by a one-pot process (hydrolysis of precursors TEOS and TEPO, polymerization) and silica matrix formation by crosslinking.

Tetraethyl orthosilicate (TEOS), triethyl phosphate (TEPO), ethanol, sodium chloride, and deionized water were mixed in a plastic beaker. The reaction was then catalyzed by the addition of hydrochloric acid and stirred to promote the hydrolysis and homocondensation of TEOS and the incorporation of TEPO into the forming polymer. Finally, triethylamine (TEA) was added to induce gelation (crosslinking) of the polymer (Figure 1). The resulting gel was aged for three days at room temperature. Subsequently, the material was dried in an oven at 60 °C, and the dried material was then washed three times using deionized water and acetone. Each washing step involved (1) dispersing the material in the solvent mixture, (2) stirring for 10 min, and (3) centrifugation to separate the solid phase. The washing cycle was repeated until the supernatant was clear, ensuring the removal of unreacted species and by-products. After the washing procedure, the material was dried again in an oven at 60 °C. Different synthesis formulations were evaluated by varying the TEOS:TEPO molar ratio and the pre-gel hydrolysis and condensation time, as summarized in Table 1. The time reported in Table 1 corresponds to the reaction period before TEA-induced gelation, whereas all gels were subsequently aged for three days at room temperature. Because several formulation variables were modified simultaneously, this experimental matrix was used as an empirical synthesis rather than as a factorial optimization of individual synthesis parameters.

### 2.3. Characterization of SG-P Adsorbent

#### 2.3.1. Textural Properties

Textural properties of the material’s surface were performed utilizing nitrogen adsorption–desorption analyses by an advanced ASAP 2020 KMP (Micromeritics, Norcross, GA, USA) system at 77 K. SG and SG-P1–6 samples of approximately 0.3 g each underwent a thermal treatment at 423 K under vacuum (10 µm Hg) for 6 h to ensure the complete desorption of surface-bound species. The specific surface area was quantified through the Brunauer–Emmett–Teller (BET) method, analyzing adsorption data across the relative pressure interval of 0 < P/P_0_ < 0.3. The total pore volume was assessed at a relative pressure of P/P_0_ = 0.995, the pore size distribution was obtained using the Barrett–Joyner–Halenda (BJH) method, with data acquired in the pressure range of 0.42 < P/P_0_ < 0.98.

#### 2.3.2. Potentiometric Titration of the SG-P Adsorbent

The functionality of the phosphate moieties in the SG-P adsorbent was determined by an automatic potentiometric titration procedure. A sample of 0.25 g of adsorbent was placed in a double-walled Pyrex vessel, mixed with 50 mL of a 0.1 N NaNO_3_ solution used as an inert electrolyte. This mixture was equilibrated at 25 °C overnight under CO_2_-free conditions by a continuous N_2_ flow to avoid dissolution of CO_2_ gas into the system. The initial pH of the sample was measured with an Orion VersaStar Pro pH meter from Thermo Scientific (Singapore) (±0.001 pH units of resolution). A single junction glass pH electrode (Orion, Ross Ultra Thode pH range: 1–13, filled with 3 M KCl) was calibrated using commercial pH buffers (buffer solutions with pH = 4.00, 7.00, and 10.00). After equilibrium was completed, a small amount of either acid or base was added to shift the pH to the initial titration pH. After this starting point was reached, titration with 0.1 N NaOH (or HNO_3_) was performed using a Dosimat 876 plus Metrohm microburet (Metrohm AG, Herisau, Switzerland), which allows for accurate dosing of titrant additions (±0.001 cm^3^). The microburet was connected to a PC terminal that administered the titrant dose. The titration process was controlled by a LabView program (LAbView 2020). To control titrant additions, the stability of pH was determined as follows: the advancement of titration was established by the restrictions permitted for pH change (ΔpH) and the time pauses at which pH values were compared (Δt). In these experiments, ΔpH was set to 0.005 pH units, Δt was set to 60 s, and Δv was set to 0.05 mL. The experiments were carried out in a pH range of 3–11 in triplicate to determine the uncertainties of pKa values. The amount of protons reacted per mass of adsorbent Q, in mmol g^−1^, was calculated from a balance on protons given by (Equation (1)):

(1) $$Q\;=\;\frac{1}{m}\left\{V_{i}(C_{a}^{i}-C_{b}^{i})+VN_{T}-(V_{i}+V)\left({\left[H^{+}\right]}_{f}-{\left[OH^{-}\right]}_{f}\right)\right\}$$

where *m* is the mass of adsorbent, *Vi*, is the initial volume of solution, *V* is the volume of titrant, $C_{a}^{i}$ and $C_{b}^{i}$ are the initial analytical concentrations of acid (or base) added initially to the system, *N_T_* is the normality of titrant, and ${\left[H^{+}\right]}_{f}$ and ${\left[{OH}^{-}\right]}_{f}$ are the actual concentrations of these ions corrected for activity coefficients from the experimentally measured values using the Davies equation [19]. The titration data were transformed into the proton isotherm (Q(pH)), and then the pKa values of the phosphate groups were calculated using the proton affinity distributions (PAD) method with the SAIEUS 2.02 program [20,21].

#### 2.3.3. FTIR Analysis

Samples of the functionalized material SG-P and SG-P-Cu after copper adsorption, as well as a sample of non-functionalized silica gel (SG), used as a control, were analyzed by Fourier-transform infrared spectroscopy (FTIR) to determine changes in the chemical composition. All measurements were performed in a Thermo Fisher Scientific Nicolet FTIR spectrometer Model iS50 equipped with an attenuated total reflectance (ATR) device. Spectra were acquired after 16 scans using a spectral width range of 500–4000 cm^−1^ and a resolution of 4 cm^−1^.

#### 2.3.4. ^29^Si and ^31^P NMR Spectroscopy

NMR measurements were performed on an InfinityPlus NMR spectrometer (Agilent Technologies, Santa Clara, CA, USA) operating at 7 T. Samples were packed into 6 mm ZrO_2_ pencil rotors and measured at a magic-angle spinning (MAS) speed of 6 kHz. A 90° pulse length of 5.0 μs and a relaxation delay of 3 s were used. Cross-polarization (CP) NMR spectra were recorded with a contact time of 5 ms. Please note that the relative intensities of signals in the CP MAS NMR spectra do not correlate with the relative amounts of the corresponding species. Detailed discussion of this point can be found elsewhere [22,23].

### 2.4. Copper Adsorption Tests

#### 2.4.1. Aqueous Solutions of Cu (II)

A 1000 mg L^−1^ Cu(II) stock solution was prepared by dissolving dry CuSO_4_·5H_2_O in deionized water. All diluted solutions were prepared by dilution of the stock solution. The concentration of Cu was determined by an atomic absorption spectrometer (Agilent 240FSAA, Agilent Technology).

#### 2.4.2. Maximum Cu(II) Uptake

The maximum Cu(II) uptake measurement was tested for all SG-P samples to select the material with the highest Cu(II) uptake performance. Each of the synthesized adsorbents (50 mg) was contacted with 1 mL of a 100 mg L^−1^ Cu(II) solution at pH 5 and stirred at 120 rpm at 303 K for 24 h. After that time, the liquid was separated, and the initial and final Cu concentrations were measured. The amount of Cu adsorbed at equilibrium was determined by the mass balance (Equation (2)):

(2) $$q=\frac{V\left(C_{0}-C_{f}\right)}{m}$$

where *q* is the amount of Cu(II) adsorbed on the surface of adsorbents (mg g^−1^), *V* is the volume of the Cu(II) solution (L), *C*_0_ and *C_f_* are the initial and final Cu(II) solution concentrations (mg L^−1^), respectively, and *m* is the mass of adsorbent used.

#### 2.4.3. Adsorption and Equilibrium Experiments

Additional adsorption kinetic and equilibrium experiments were performed to further characterize the Cu(II) adsorption behavior of the synthesized materials. The experimental procedures, nonlinear regression analyses, kinetic modeling, equilibrium isotherm modeling, and the corresponding results are described in Section S3 of the Supplementary Materials. Figure S3 and Table S4 present the experimental kinetic data, nonlinear model fittings, and fitted kinetic parameters, whereas Figure S4 and Table S5 present the experimental equilibrium isotherm, nonlinear isotherm fittings, and fitted isotherm parameters.

### 2.5. EDX Elemental Analysis

Scanning electron microscopy (SEM) coupled with energy dispersive X-ray spectroscopy (EDX) was used to analyze the elemental composition of the SG-P adsorbent before and after the Cu(II) uptake process. The analyses were carried out with a JEOL JSM6610LV (JEOL Mexico, Ciudad de México, Mexico) at an accelerating voltage of 20.0 kV, using a tungsten filament. This setup allowed for high-resolution imaging and accurate EDX detection, providing essential information about the changes in elemental distribution and structural features of the adsorbent.

#### Cu Adsorption–Desorption Process Using a Phosphate Reload System

**Figure 2:**
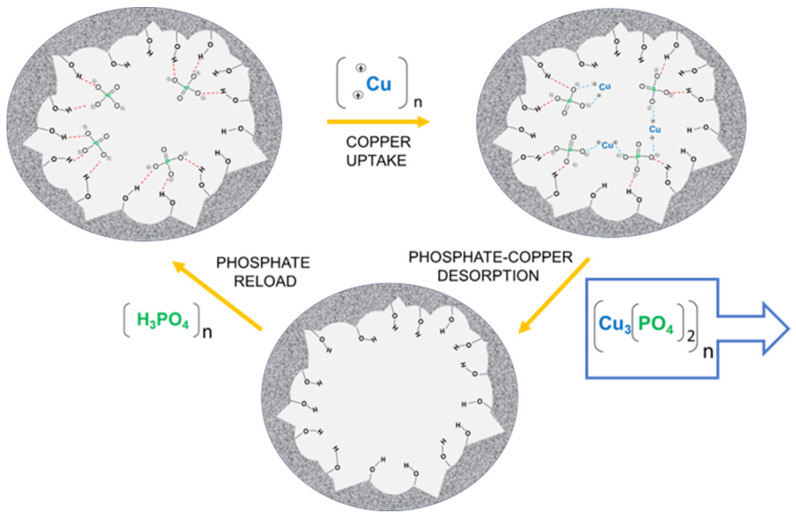
Schematic representation of the functional recycling system of SG-P in three steps: copper uptake by a copper solution, phosphate–copper desorption in acid media, and phosphate reloading process using phosphoric acid.

The reuse of phosphate-functionalized silica (SG-P) focused on evaluating at least three phosphate-reloading and Cu(II) uptake cycles through consecutive adsorption–desorption processes. After Cu(II) uptake, the resulting SG-P-Cu material was submitted to a desorption treatment using a 0.02 M HCl solution for 24 h at 303 K under continuous stirring at 120 rpm. This acid treatment was used to remove both the adsorbed Cu(II) ions and the phosphate moieties weakly retained within the silica cavities. The solid was subsequently separated from the acid solution by filtration, washed three times with distilled water to eliminate residual acid, and dried at 60 °C for 2 h. The desorbed sample was then analyzed by SEM-EDX to confirm the effective removal of phosphorus and copper species. Following the desorption step, the silica gel matrix was reloaded with phosphate groups by contacting it with a 0.01 M H_3_PO_4_ solution under identical conditions (303 K, 120 rpm, 24 h). After reloading, the material was filtered, washed with distilled water, and dried at 60 °C. Again, the successful reincorporation of phosphate moieties within the silica structure was verified by SEM-EDX. This functional recycling system was designed to evaluate the ability of the silica matrix to recover its functional sites by introducing fresh phosphate fractions from affordable phosphoric acid and maintain its Cu(II) adsorption capacity. This information will provide important data on the behavior of the SG-P material after repeating multiple cycles of functional regeneration and adsorption–desorption processes (Figure 2).

## 3. Results and Discussion

### 3.1. Textural Properties of SG and SG-P Adsorbents

**Figure 3:**
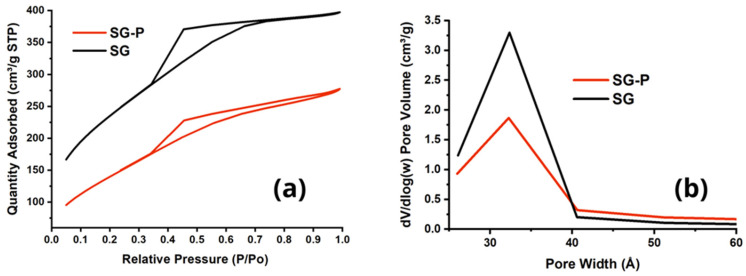
(**a**) Nitrogen adsorption–desorption isotherms of SG and SG-P, and (**b**) pore size distribution of SG and SG-P. The changes related to the decrease in isotherm profiles and pore size distribution of SG versus SG-P are attributed to the presence of phosphate groups in the silica gel matrix.

The synthesis conditions produced changes in the textural properties of SG-P1–SG-P6, as summarized in Table S1. For the formulations prepared at a TEOS:TEPO molar ratio of 1:1, increasing the pre-gel hydrolysis and condensation time from 2 to 24 h slightly decreased the BET surface area from 498.15 to 488.56 m^2^ g^−1^, while the pore volume increased from 0.1785 to 0.6204 cm^3^ g^−1^, and the mean pore diameter increased from 42.40 to 47.80 Å. For the TEOS:TEPO ratio of 2:1, increasing the reaction time from 2 to 72 h decreased the BET surface area from 586.68 to 515.12 m^2^ g^−1^ and the pore volume from 0.4726 to 0.1474 cm^3^ g^−1^, whereas the mean pore diameter increased from 31.26 to 36.58 Å. For the formulations prepared at a TEOS:TEPO ratio of 1:2, increasing the reaction time from 2 to 72 h decreased the BET surface area from 522.61 to 382.17 m^2^ g^−1^, slightly increased the pore volume from 0.3656 to 0.3856 cm^3^ g^−1^, and increased the mean pore diameter from 39.22 to 44.29 Å. These results indicate that longer pre-gel reaction times generally decreased the BET surface area and increased the mean pore diameter, whereas the pore volume did not follow a consistent trend. However, because the TEA content and other synthesis variables were not maintained constant in all formulation pairs, the observed changes cannot be attributed exclusively to either the TEOS:TEPO molar ratio or the reaction time. In addition, total phosphorus loading was not quantitatively determined for all six formulations; therefore, a direct quantitative relationship between the synthesis conditions and phosphate loading cannot be established from the present dataset. SG-P5, hereafter referred to as SG-P, was selected for subsequent characterization and equilibrium experiments because it exhibited a suitable combination of mesoporosity and Cu(II) uptake. In the kinetic, SG-P5 showed the highest experimental uptake among the phosphate-containing formulations evaluated. Figure 3a shows the nitrogen adsorption–desorption isotherms of the functionalized material SG-P and silica gel (SG) (used as a control sample). Both materials exhibit type IVa isotherms with H3-type hysteresis loops, characteristic of mesoporous solids according to the IUPAC classification [24]. This behavior indicates the presence of slit-shaped pores where capillary condensation occurs. Compared with SG, the SG-P material shows a significantly lower nitrogen uptake, reflecting a decrease in surface area and pore volume upon functionalization with phosphate groups. This reduction is attributed to the partial occupation of mesopores by phosphate moieties introduced during the sol–gel synthesis, which decreases the accessibility of the porous network. Figure 3b displays the pore size distribution curves. Both materials exhibit mesopores in the range of 20–40 Å. SG-P and SG show a main peak centered at ~32 Å, confirming a mesoporous framework with similar pore size distribution. However, the intensity of the peak for SG is higher, indicating a greater pore volume contribution from this size range. In contrast, SG-P shows a lower peak intensity, consistent with partial pore blocking or reduced accessibility after the introduction of phosphate moieties. The corresponding textural parameters obtained from BET analysis are summarized in Table 2. SG exhibits a specific surface area of 849.5 m^2^/g and a pore volume of 0.456 cm^3^ g^−1^. After functionalization, SG-P presents a reduced surface area of 522.6 m^2^/g and a pore volume of 0.366 cm^3^ g^−1^, while the mean pore diameter increases slightly from 36.2 to 39.2 Å. These results confirm that the introduction of phosphate moieties decreases the available surface area but modifies the pore structure toward slightly larger mesopores. Such structural modifications are relevant for adsorption applications since they indicate that the phosphate groups, although partially reducing surface accessibility, remain available within the porous framework to interact with Cu(II) ions in aqueous solution. This structural evidence supports the rationale for testing SG-P as a functionalized adsorbent in comparison with pristine silica.

### 3.2. Potentiometric Titrations

**Figure 4:**
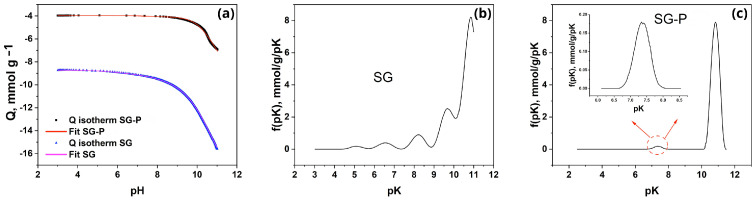
Titration results of two solid adsorbents. (**a**) Proton binding isotherms of SG-P and SG samples. Solid lines indicate the fit of the experimental points to the integral adsorption equation used in the SAIEUS software; (**b**) pK distribution for pristine silica sample SG; (**c**) pK distribution for SG-P adsorbent.

**Figure 5:**
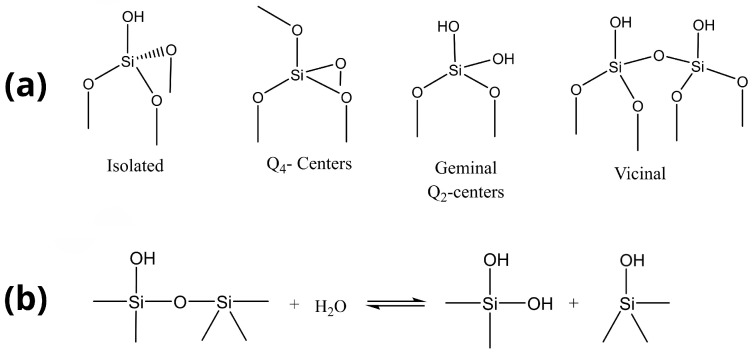
(**a**) Typical scenarios for silanol groups on the surface of amorphous silica (isolates, center, geminal, and vicinal species), and (**b**) reactions occurring on the silica/solution as pH changes.

With the aim of comparing the changes on the silica surface in the phosphate functional group and during the Cu(II) ions adsorption process identified by the potentiometric titrations, a control sample was synthesized by the sol–gel method and called SG. The crude data generated by the potentiometric titrations are shown in Figure 4, and the corresponding parameters can be found in Table 3. In Figure 4a, the data are plotted as proton binding isotherms (Q vs pH) for the two samples SG and SG-P. Positive values for Q (pH) indicate proton uptake and negative values show proton release (i.e., the more negative the more acidic). The two samples have Brꬾnsted acidic characters. However, based on the magnitude of the Q values, it is seen that the SG sample presents an increased acidity compared to the SG-P sample. This is due to the presence of more OH groups on the surface of SG-P, according to the reaction: $-S-OH+OH\;↔\;-SO^{-}+\;H_{2}O$. In the case of SG, the high degree of acidity is due to the more abundant charged groups, as indicated by the reaction: $Si{OH}_{2}^{+}↔SiOH+\;H^{+}$. Thus, the overall reaction occurring during the titration course is given by: $SOH_{2}^{+}↔SOH↔SO^{-}$, where S denotes a site on the surface of the adsorbent. Figure 4b shows a region below pH 7 where two small peaks are present, at pKa values 5.09 and 6.56. The value at pK 5.09 (0.09 OH nm^−2^) corresponds to the out-of-plane geminal silanols, and this value is associated with the H-bond strengths that the silanols form with the foremost adsorbed water layer, i.e., it corresponds to the case of strong H-bonds (out-of-plane) [25]. The value at pKa 6.56 (0.23 OH nm^−2^) corresponds to the presence of isolated silanols on the surface of SG [26]. The different types of silanol groups are shown in Figure 5a. Above pH 7, where proton release starts taking place, the *f* (pK) spectrum shows ionization of geminal concave silanols at pKa 8.23 (0.49 OH nm^−2^) [25] followed by strong proton release caused by the separation of siloxane groups at pKa 9.67 (1.53 OH nm^−2^) [27], after which dissociation of silanol groups occurs and dissolution of silica takes place at pKa 10.84. This process is illustrated in Figure 5b. Our values agree with reports from the literature [27]. The density of total active silanols is 2.34 OH nm^−2^. This result agrees with reports from the literature where a range of 0.5–8 group nm^−2^ has been established in the case of amorphous silica and depends on the synthesis and pretreatment procedures [28,29,30].

When the SG sample was functionalized with phosphate moieties (SG-P), surface chemistry was significantly changed. The presence of isolated hydroxyl groups bound to phosphorus (P-OH) [31] is demonstrated by the value of pKa 7.1 ± 0.28 (0.11 OH nm^−2^) (Figure 4c inset and Table 3). Hydrolysis of the triethyl phosphate moieties has been reported in the literature [16]. The absence of peaks at pH below 7 is notable in the SG-P sample, indicating that hydrolysis and condensation reactions were carried out almost to completion during the synthesis.

### 3.3. FTIR Analysis of SG and SG-P Before and After Cu(II) Uptake

**Figure 6:**
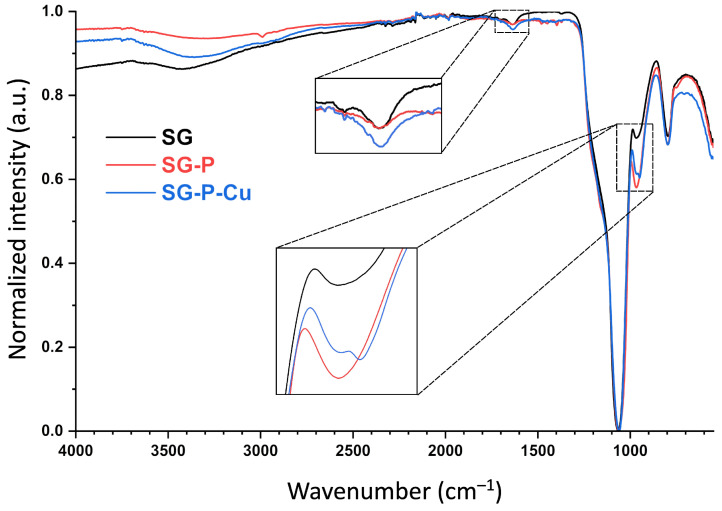
FTIR spectra of SG, SG-P, and SG-P-Cu (inset: band splitting into the 1040–900 cm^−1^ region due to Cu(II)–phosphate interaction and the 1700–1600 cm^−1^ associated with adsorbed water).

The FTIR spectra of SG (black), SG-P (red), and SG-P-Cu (blue) samples are shown in Figure 6. The SG spectrum exhibits a single broad signal at 3300 cm^−1^ due to O-H stretching vibrations of silanol groups, which is corroborated by the small signal near 966 cm^−1^ of OH bending vibration mode. The signal at 1060 cm^−1^ with the highest intensity corresponds to the Si–O–Si and Si-OH stretching vibrations of the silica gel matrix. In the case of the SG-P spectrum, these signals described for the SG sample are also present. However, the presence of P–O and P=O vibrations from the hydrolysis of the triethyl phosphate (TEPO) molecules arose in the SG-P spectrum by means of the increased signal at approximately 967 cm^−1^ (defined already as OH bending vibrations), indicating extra contributions due to possible signals overlapping (inset region 1040–900 cm^−1^). In addition, the absence of frequency shifting of the main Si–O–Si signal (1060 cm^−1^) could be associated with the lack of Si–O–P covalent bonds. On the other hand, this signal behavior could support the idea of phosphate confinement in silica gel cavities stabilized by ionic or hydrogen bonds that agree with previous reports on similar silica–phosphate materials [32,33].

**Figure 7:**
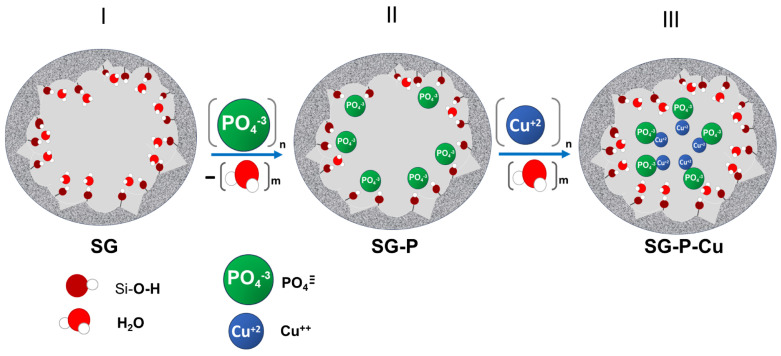
Possible scenarios of water molecules replacement from the SG surface (**I**) by phosphate moieties stabilization (SG-P) (**II**) and water re-adsorption after the copper uptake by phosphates (SG-P-Cu) (**III**). This mechanism demonstrates the non-covalent association of phosphate groups to the silica gel matrix before and after the copper adsorption.

Furthermore, after Cu(II) exposure, the spectrum of SG-P-Cu shows a perceptible change in the P-O signal. This band at 967 cm^−1^ becomes broader and splits into two, with a new signal emerging near 949 cm^−1^. The frequency of this new signal is consistent with previous observations of Cu–O–P interactions in phosphate-silicate materials, where new components appear around 944 cm^−1^ upon metal incorporation [34]. Therefore, the observed band splitting and intensity increase are attributed to the coordination of Cu(II) ions with oxygen atoms from the phosphate groups, forming the P–O···Cu interactions. Similar behavior has been associated with phosphate vibrations overlapping the Si–O–Si stretching region, commonly observed in the 1000–1200 cm^−1^ range for hybrid silicate materials [32]. In addition, all FTIR spectra of the samples depicted the classical adsorbed water signal around 1640 cm^−1^ but with different signal intensities (inset region between 1700 and 1600 cm^−1^). Here, the sample of SG-P in comparison with the original SG or the SG-P-Cu showed the least intense signal (also corroborated by the decrease in OH broad signal around 3300 cm^−1^) that could be interpreted as the phosphate moieties replacing the water molecule sites on the silica gel surface. This observation supports the stabilization of phosphate through hydrogen bonding with silanol groups on the material surface and the P-Cu desorption mechanism as proposed in the scenarios (I to III) of Figure 7.

### 3.4. ^29^Si and ^31^P NMR Analyses

**Figure 8:**
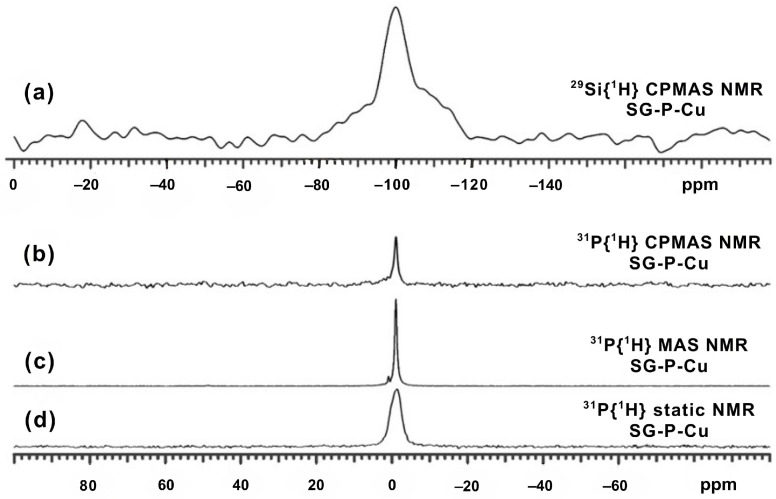
Solid-state NMR spectra of SG-P-Cu: (**a**) ^29^Si{^1^H} CPMAS with an intense signal of Q^3^ species (corresponding to isolate and vicinal species in Figure 5a, (**b**) ^31^P{^1^H} CPMAS depicts a single phosphorus signal at −1 ppm related to phosphate chemical shift, (**c**) ^31^P{^1^H} MAS with same signal at −1 but with higher intensity due to the rotational diffusion, and (**d**) ^31^P{^1^H} static shows a slight signal broadening, demonstrating the small chemical anisotropy that is also related to rotational diffusion and confirms the [PO_4_]^3−^ species.

Figure 8a shows the ^29^Si{^1^H} CPMAS NMR spectrum of SG-P-Cu. The dominant signal at −101 ppm and two minor signals at −92 ppm and −111 ppm are assigned to Q^3^, Q^2^, and Q^4^ species of the polymeric silica matrix, respectively. The high intensity of the Q^3^ signal reflects the high concentration of silanol groups on the surface of the silica gel cavities bearing the phosphate–Cu complexes, as proposed in Figure 7. In contrast, the absence of any signals in the −50 to −70 ppm region indicates that the sample does not contain functional groups covalently attached to the silica surface [15].

Figure 8b,c show the ^31^P{^1^H} CPMAS and ^31^P{^1^H} MAS NMR spectra of SG-P-Cu. Both spectra exhibit a signal at −1.0 ppm. However, the intensity of this signal in the ^31^P{^1^H} MAS NMR spectrum is much higher than in the CP spectrum, indicating that the rotational diffusion of the corresponding phosphorus species occurs on the millisecond timescale. The signal becomes only slightly broader in the static ^31^P{^1^H} NMR spectrum, Figure 8d. Therefore, the chemical shift anisotropy is either small or effectively reduced by rotational diffusion. Both the chemical shift value and the observed rotational diffusion support the assignment of this signal to [PO_4_]_3_− species. In addition, copper adsorption does not significantly alter the ^29^Si or ^31^P NMR spectra of the silica matrix. The absence of changes in the ^29^Si spectrum is expected, since copper does not chemically react with the silicate framework. For ^31^P, any changes are likely averaged out by rapid exchange, on the NMR timescale, between different non-covalently bound copper–phosphorus complexes.

### 3.5. Copper Adsorption–Desorption Performance of SG-P Material (A Preliminary Approach)

**Figure 9:**
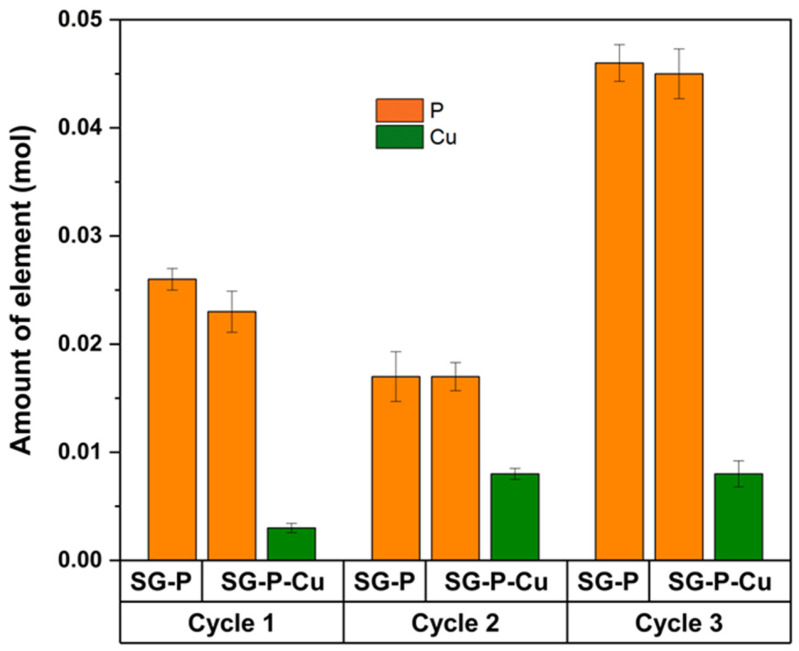
Molar amount of phosphorus and copper determined by SEM–EDX for SG-P and SG-P-Cu samples in three consecutive Cu(II) adsorption cycles. Each cycle is divided by the initial amount of phosphorus adsorbed by the molded silica gel polymer and the amount of phosphorus and copper after Cu(II) adsorption.

SEM–EDX was applied to monitor the presence and the amount of phosphorus and copper in the material as well as their changes during the Cu(II) uptake, desorption, and ligand-reloading cycles. Figure 9 summarizes the molar amounts of phosphorus and copper determined on the surface of the SG-P and SG-P-Cu samples, before and after each adsorption step, for three consecutive cycles. The values in the bar chart were obtained by converting the SEM–EDX weight percentages (Table S2) into moles of element per sample, allowing a direct comparison of the relative abundance of P and Cu(II) associated with the adsorbent at each stage. In the first cycle, the freshly synthesized SG-P material contains exclusively phosphorus (0.8 wt%, 0.026 mol). This result confirms the incorporation of phosphate groups into the silica matrix and the absence of residual metal in the material. After contact with the Cu(II) solution, the SG-P-Cu sample shows a detectable amount of copper at 0.20 wt% (0.003 mol) and a slight decrease in the phosphorus content (0.7 wt%, 0.023 mol). These changes indicate that not all phosphate groups participate in the coordination of Cu(II) ions, and an excess of phosphates remains free. This P/Cu molar ratio of 8:1, in the first adsorption step, can be regulated by the availability and accessibility of the phosphate groups. Afterward, in the desorption process, the washing with acid removes the P-Cu complex from the surface, as shown by SEM–EDX data of the desorbed material, where phosphorus and copper are no longer detected. These results, provided in Table S2 of the Supporting Information (SG, P–Cu desorbed), confirm that the SG-P bond is non-covalent as was already observed by FTIR and ^31^P-NMR analyses. Now, to restore the adsorption capacity, the silica matrix was reloaded with a more affordable source of phosphate groups, such as H_3_PO_4_, before the second Cu(II) uptake. After this reloading step (cycle 2), the SG-P sample confirms the presence of phosphorus (0.53 wt%, 0.017 mol), and the sample after copper adsorption (SG-P-Cu) shows a Cu amount of 0.51 wt% corresponding to 0.008 mol, this value represents a P/Cu molar ratio close to 2:1. This behavior differs from the first cycle and suggests that, after phosphorus reloading step, the phosphate groups are better organized in cavities of the silica matrix where copper ions are more accessible for metal coordination, improving the performance of the ligand in the regenerated material. In the third cycle, the scenario changes markedly. A second reloading with H_3_PO_4_ produces a strong increase in the phosphorus content of SG-P (1.43 wt%, 0.046 mol) compared with cycles 1 and 2. However, the amount of copper adsorbed in the SG-P-Cu sample (0.53 wt%, 0.008 mol) remains similar to that observed in the previous adsorption process (Cycle 2). The lack of proportionality between the increase in P and the Cu(II) uptake indicates that a significant fraction of the phosphate detected by SEM–EDX is not fully available for coordination. A reasonable explanation is that the repeated treatment with acid in the desorption step and even the phosphoric acid in the recharging process promotes some hydrolysis erosion that restructures the surface of the silica matrix. This generates increased surface area and additional phosphate-rich regions partially blocked or sterically hindered for copper accessibility. This interpretation was corroborated by nitrogen adsorption–desorption measurements (BET data) of silica gel treated with phosphoric acid (Figures S1 and S2, Table S3).

On the other hand, these new functionalized regions contribute to the overall phosphorus amount but do not provide an equivalent number of accessible binding sites for Cu(II). In summary, the SEM-EDX results demonstrate the rechargeable functional potential of the SG-P hybrid adsorbent and the clear preference of phosphate groups by Cu(II) ions, as well as the total removal of the P-Cu complex during the desorption stage. In addition, molded cavities in the polymer can be repurposed using a cheaper phosphorus source, such as H_3_PO_4_, restoring the copper adsorption capacity.

Furthermore, although the functional phosphate is not covalently grafted to the silica gel matrix, the hydrogen bond interactions between the silanol on the surface of the polymer material and the phosphorus ligands appear to be stable enough for the adsorption process, as was also observed by a potentiometric titration experiment.

As a first approximation, through this SEM-EDX data, the reuse of the silica polymer center in the rechargeable system and its performance after three cycles is clearly demonstrated. This data provided a preliminary approximation for projecting profitability and potential long-term performance. However, it is important to highlight that the main objective of this work was to demonstrate a new perspective in the design of functional materials on the recyclability of the functional system in molded polymer cavities. In addition, through cost-effective active functional sources such as phosphoric acid.

On the other hand, this molecular view of adsorption is hardly described in most of the published literature on new materials developed for the removal of heavy metals from aqueous media. Conversely, a complete study of the adsorption efficiency performance of this polymeric material using kinetic and equilibrium models, as well as more comprehensive testing of its reusability, is beyond the scope of the present work. However, all of them are planned as the next logical step in this research.

### 3.6. Comparison with Recent Functionalized Silica and Ion-Imprinted Adsorbents

Table 4 compares the present material with recently reported silica-based, phosphorus-containing, and ion-imprinted adsorbents. However, adsorption capacities cannot be compared directly because the studies used different target ions and experimental conditions. The SG-P material showed a lower Cu^2+^ uptake than some covalently functionalized or ion-imprinted adsorbents [33,34,35,36,37,38]. However, its main advantage is not a high adsorption capacity, but its rechargeable phosphate system. The silica matrix acts as a stable porous support, while the phosphate groups act as replaceable ligands. During acid treatment, the Cu^2+^–phosphate species are removed. The phosphate-containing functional sites are then restored by treatment with H_3_PO_4_. This process differs from conventional regeneration methods, in which the functional ligand remains permanently attached to the adsorbent and only the captured metal ion is removed. Recent phosphate and phosphonate-functionalized silica materials have shown high affinity, selectivity, or magnetic recovery [33,34,35,36,37,38]. In contrast, the present study focuses on replacing the phosphate ligands that functionalize the printed cavities, while preserving the same porous silica scaffold for phosphate recognition.

Although the SG-P material does not compete in metal adsorption capacity with classical hybrid materials with covalently bonded ligands, this work represents the first step towards the development of new rechargeable materials that can be improved after further research. Moreover, phosphate-functionalized silica (P-SG) is presented as a cost-effective, recyclable adsorbent thanks to its ability to regenerate phosphate-formed cavities using conventional phosphoric acid, without losing its copper adsorption capacity. Finally, this finding opens up the possibility of a new and affordable option for treating water streams contaminated with heavy metal cations such as copper.

## 4. Conclusions

A hybrid adsorbent of silica gel polymer and phosphate functional groups (SG-P) was successfully synthesized by one-pot sol–gel reaction. Molecular imprinting with triethyl phosphate (TEPO) was used to create functional phosphorus cavities in the silica matrix. Reduction in the textural properties of specific area and pore volume in the SG-P in comparison with unfunctionalized SG was attributed to the presence of phosphate functional groups in the adsorbent. FTIR and ^29^Si and ^31^P NMR spectroscopy were performed to inspect the chemical and structural composition of the material. The ^29^Si NMR analysis of SG-P-Cu indicates that the phosphate ligands are stabilized within the cavities through interactions with surface silanol groups. This is supported by ^31^P NMR analysis and corroborated by potentiometric titration experiments. Furthermore, changes in the signal intensity in the FTIR spectra due to water adsorbed onto the material surface before and after the Cu(II) uptake also indicate the phosphate stabilization and the dynamics of the P-Cu complex release from the silica gel surface and its elimination in the desorption step. Copper adsorption experiments by the SG-P materials showed removal performance by phosphate-metal complexation. The Tóth isotherm model provided the best overall fit, suggesting adsorption on a heterogeneous surface, which is consistent with the structural heterogeneity. The recyclable ligand system of the adsorption–desorption and phosphate regeneration maintains a similar uptake capacity after the third cycle. To our knowledge, this investigation proposes the first approach to a recyclable polymeric adsorbent by designing functionalized silica cavities using molecular imprinting with active non-covalent functional ligands. Furthermore, these cavities have the advantage of being able to be replenished with an affordable phosphate source without losing their metal absorption capacity.

## Figures and Tables

**Table 1:** Composition of the empirical synthesis used to prepare SG and SG-P1–SG-P6 at different TEOS:TEPO molar ratios and pre-gel hydrolysis and condensation times.

	Molar Ratios								
Sample	TEOS	TEPO	EtOH	NaCl	H_2_O	HCl	TEA	TEOS:TEPO	Pre-Gel Hydrolysis and Condensation Time (h)
SG-P1	1	1	7	0.010	7	0.018	0.72	1:1	2
SG-P2	1	1	7	0.010	7	0.018	0.50	1:1	24
SG-P3	2	1	11	0.015	11	0.018	0.72	2:1	2
SG-P4	2	1	11	0.015	11	0.018	0.58	2:1	72
SG-P5	1	2	10	0.015	10	0.012	0.43	1:2	2
SG-P6	1	2	10	0.015	10	0.012	0.43	1:2	72
SG	1	-	4	0.010	4	0.018	0.72	1:0	2

**Table 2:** Textural parameters related to specific surface area, pore volume mean, and pore size distribution of SG and SG-P adsorbents. The decrease in these values in the SG-P sample compared to SG is associated with the presence of phosphate on the surface and in the cavities.

Sample	BET Surface (Area m^2^ g^−1^)	Mean Pore Volume (cm^3^ g^−1^)	Pore Size (Å)
SG	849.50	0.456	36.2
SG-P	522.61	0.366	39.2

**Table 3:** Titration parameter results of the SG and SG-P samples. The pka data, Brønsted acid ligand density, and its assignment for pristine (SG) and functionalized (SG-P) samples are shown.

Peak No.	SG				Reference
	pk*_a_*	Pk. Area	Brꬾnsted Acid Ligand Density, OH/nm^2^	Assignment	
1	5.09	0.12	0.09	Geminal silanols out-of-plane	[25]
2	6.56	0.33	0.23	SiOH = H^+^ + SiO^−^	[26]
3	8.23	0.69	0.49	Geminal concave OH	[25]
4	9.67	2.16	1.53	Isolated silanols pK	[27]
5	10.84	5.78	-----	Silica dissolution	[27]
Total OH sites:			2.34		
SG-P					
1	7.1 ± 0.28	0.1	0.11	Isolated OH of P-OH	This work
2	10.8 ± 0.02	4.96	-----	Silica dissolution	Contescu et al. 1995 [27]

**Table 4:** Comparison of the present phosphate-rechargeable silica with representative recent silica-based, phosphorus-containing, and ion-imprinted adsorbents.

Material (Ref.)	Functionalization/Synthesis Strategy	Ion and Adsorption Performance	Regeneration/Reuse	Highlight
SG-P (this work)	One-pot TEOS/TEPO sol–gel synthesis; non-covalently retained phosphate; separate H_3_PO_4_ reloading	Cu(II): qmax, exp = 2.09 mg g^−1^; Tóth qmax = 2.06 ± 0.05 mg g^−1^	Three adsorption–desorption–reloading cycles; 0.02 mol L^−1^ HCl followed by 0.01 mol L^−1^ H_3_PO_4_	Replaceable ligand fraction within a reusable silica scaffold. The first experimental approach reported
SiN-imd-py [35]	Post-synthesis grafting of a Schiff-base ligand onto mesoporous silica	Cu(II): Langmuir qmax = 103.52 mg g^−1^	Five cycles; uptake decreased from 97.17 to 88.43 mg g^−1^	High Cu(II) capacity and selectivity with a permanently grafted ligand
SiO_2_–Ti–P–Zr [36]	Layer-by-layer grafting of zirconium–titanium phosphate onto SBA-15-type silica	Ni(II): Langmuir qmax = 50.1 mg g^−1^	Adsorption cycling not reported; <0.4% Ni release after 14 d in 0.1 mol L^−1^ HCl	Phosphate-containing silica with strong binding and acid stability
SMS–Ph [37]	Mesoporous silica prepared using sodium metasilicate and phosphoric acid	U(VI): reported qe = 820.7 mg g^−1^ near pH 8	Regeneration not reported	Low-cost phosphorus source and high reported selectivity; comparison limited by U(VI) speciation
MMS–PP [38]	Post-grafted phosphonate groups on magnetic mesoporous silica	La(III)/REEs: 13.5 mg g^−1^ La in 50 mmol L^−1^ citrate	Multiple cycles; 70–95% REE recovery from real waste extracts	Magnetic separation and operation in complex citrate-assisted extracts
Pore-expanded phosphate silica [39]	One-pot synthesis using HDEHP as a co-template, followed by pore expansion	U(VI): Langmuir qmax = 136 mg g^−1^ at pH 4	Reusable material; particles recoverable by low-g centrifugation or sedimentation	Large recoverable microspheres with phosphate functionality
IIP-Cu [40]	Cu(II)-templated ion-imprinted polymer followed by template removal	Cu(II): Langmuir qmax = 159 mg g^−1^; 98.7% removal at optimum pH	High desorption efficiency; 90.6% Cu(II) removal from real brine	Ion-imprinted recognition sites and validation with competing ions

Note: Adsorption capacities are not directly comparable because target ions, solution pH, initial concentration, adsorbent dosage, temperature, ionic composition, and equilibrium criteria differ. Reported capacities at conditions where metal hydrolysis or precipitation may occur should be interpreted cautiously.

## Data Availability

The data presented in this study are available upon request from the corresponding authors, J.B.-C and R.M-G.

## Supplementary Materials

The following supporting information can be downloaded at: https://www.mdpi.com/article/10.3390/polym18141759/s1, Figure S1: Nitrogen adsorption–desorption isotherms of SG and SG treated with H_3_PO_4_; Figure S2: Pore size distribution of: SG and SG treated with H_3_PO_4_; Figure S3: Experimental adsorption kinetics of Cu(II) on phosphate-functionalized silica materials synthesized with different TEOS:TEPO molar ratios and nonlinear fitting to different kinetic models: (a) pseudo-first-order (PFO), (b) pseudo-second-order (PSO), (c) intraparticle diffusion (Weber-Morris), and (d) Avrami; Figure S4: Experimental Cu(II) adsorption isotherm for the selected phosphate-functionalized silica together with nonlinear fitting using different adsorption isotherm models: (a) Langmuir, (b) Freundlich, (c) Tóth, (d) Sips (Langmuir–Freundlich), (e) Temkin, and (f) S-shape model; Table S1: Textural surface parameters of SG-P adsorbents; Table S2: Quantitative analysis of the elemental composition by weight of SG-P and SG-P-Cu, collected from different areas by SEM-EDX method; Table S3: Textural surface parameters of SG and SG treated with H_3_PO_4_; Table S4: Kinetic parameters obtained by nonlinear regression using the Levenberg–Marquardt algorithm implemented in OriginPro 2021; Table S5: Isotherm parameters obtained by nonlinear regression using the Levenberg–Marquardt algorithm implemented in OriginPro 2021.

## References

[1] Dong D., van Oers L., Tukker A., van der Voet E. (2020). Assessing the Future Environmental Impacts of Copper Production in China: Implications of the Energy Transition. J. Clean. Prod..

[2] Ahmed E.M., Isawi H., Morsy M., Hemida M.H., Moustafa H. (2023). Effective Nanomembranes from Chitosan/PVA Blend Decorated Graphene Oxide with Gum Rosin and Silver Nanoparticles for Removal of Heavy Metals and Microbes from Water Resources. Surf. Interfaces.

[3] Choi W., Lee H.-J. (2022). Nanostructured Materials for Water Purification: Adsorption of Heavy Metal Ions and Organic Dyes. Polymers.

[4] Wang B., Lan J., Bo C., Gong B., Ou J. (2023). Adsorption of Heavy Metal onto Biomass-Derived Activated Carbon: Review. RSC Adv..

[5] Al Nawah J.Y., El-Khouly A.S. (2025). Characterization and Adsorption Behavior of Newly Synthesized Aminated Cellulose with Jeffamine EDR148 Towards Ni(II), Cu(II), and Pb(II) Heavy Metal Ions. Polymers.

[6] Ciobanu R., Bucatariu F., Mihai M., Teodosiu C. (2024). Silica-Based Composite Sorbents for Heavy Metal Ions Removal from Aqueous Solutions. Polymers.

[7] Brinker C.J., Scherer G.W. (1990). Sol–Gel Science: The Physics and Chemistry of Sol–Gel Processing.

[8] Gomez-Gonzalez S.E., Carbajal-Arizaga G.G., Manríquez-González R., De la Cruz-Hernandez W., Gomez-Salazar S. (2014). Trivalent Chromium Removal from Aqueous Solutions by a Sol–Gel Synthesized Silica Adsorbent Functionalized with Sulphonic Acid Groups. Mater. Res. Bull..

[9] Hench L.L., West J.K. (1990). The Sol-Gel Process. Chem. Rev..

[10] Badillo-Camacho J., Gutiérrez-Ortega J.A., Shenderovich I.G., Velázquez-Galván Y.G., Manríquez-González R. (2025). Monitoring Lead–Phosphorus Interactions Through 31P-NMR Used as a Sensor in Phosphine Functionalized Silica Gel Adsorbent. Gels.

[11] Gutiérrez-Ortega J.A., Gómez-Salazar S., Shenderovich I.G., Manríquez-González R. (2020). Efficiency and Lead Uptake Mechanism of a Phosphonate Functionalized Mesoporous Silica through P/Pb Association Ratio. Mater. Chem. Phys..

[12] Hernández-Velázquez P.I., Gutiérrez-Ortega J.A., Carbajal-Arizaga G.G., Manríquez-González R., la Cruz-Hernández W.D., Gómez-Salazar S. (2019). Hybrid Functionalized Phosphonate Silica: Insight into Chromium Removal Chemistry from Aqueous Solutions. J. Mex. Chem. Soc..

[13] Moran-Salazar R.G., Carbajal-Arizaga G.G., Gutierréz-Ortega J.A., Badillo-Camacho J., Manríquez-González R., Shenderovich I.G., Gómez-Salazar S. (2023). As(V) Removal from Aqueous Media Using an Environmentally Friendly Zwitterion L-Cysteine Functionalized Silica Adsorbent. Chem. Eng. Sci..

[14] Quirarte-Escalante C.A., Soto V., De La Cruz W., Porras G.R., Manríquez R., Gomez-Salazar S. (2009). Synthesis of Hybrid Adsorbents Combining Sol-Gel Processing and Molecular Imprinting Applied to Lead Removal from Aqueous Streams. Chem. Mater..

[15] Rodríguez-De-La-Peña S., Gómez-Salazar S., Gutiérrez-Ortega J.A., Badillo-Camacho J., Peregrina-Lucano A.A., Shenderovich I.G., Manríquez-González R. (2022). Novel Silica Hybrid Adsorbent Functionalized with l -Glutathione Used for the Uptake of As(V) from Aqueous Media. Ind. Eng. Chem. Res..

[16] Bueno O.M.V.M., Herrera C.L., Bertran C.A., San-Miguel M.A., Lopes J.H. (2021). An Experimental and Theoretical Approach on Stability towards Hydrolysis of Triethyl Phosphate and Its Effects on the Microstructure of Sol-Gel-Derived Bioactive Silicate Glass. Mater. Sci. Eng. C.

[17] Shenderovich I.G. (2013). Effect of Noncovalent Interactions on the 31P Chemical Shift Tensor of Phosphine Oxides, Phosphinic, Phosphonic, and Phosphoric Acids, and Their Complexes with Lead(II). J. Phys. Chem. C.

[18] Vilas Bôas da Rocha G., Henrique Lopes J., Roche V., Moreira Jorge A., Riva R., Capella de Oliveira A., Nagle Travessa D. (2022). An Innovative Strategy for Bioactivation of β-Ti12Mo6Zr2Fe Alloy Surface by Dip-Coating Method with Potential Application in the Biomedical Field. Appl. Surf. Sci..

[19] Golterman H., Stumm W., Morgan J.J. (1982). Aquatic Chemistry. An Introduction Emphasizing Chemical Equilibria in Natural Waters. J. Ecol..

[20] Jagiello J. (1994). Stable Numerical Solution of the Adsorption Integral Equation Using Splines. Langmuir.

[21] Jagiełło J., Bandosz T.J., Schwarz J.A. (1994). Carbon Surface Characterization in Terms of Its Acidity Constant Distribution. Carbon.

[22] Shenderovich I.G. (2020). For Whom a Puddle Is the Sea? Adsorption of Organic Guests on Hydrated MCM-41 Silica. Langmuir.

[23] Shenderovich I.G., Limbach H.-H. (2021). Solid State NMR for Nonexperts: An Overview of Simple but General Practical Methods. Solids.

[24] Thommes M., Kaneko K., Neimark A.V., Olivier J.P., Rodriguez-Reinoso F., Rouquerol J., Sing K.S.W. (2015). Physisorption of gases, with special reference to the evaluation of surface area and pore size distribution (IUPAC Technical Report). Pure Appl. Chem..

[25] Pfeiffer-Laplaud M., Costa D., Tielens F., Gaigeot M.-P., Sulpizi M. (2015). Bimodal Acidity at the Amorphous Silica/Water Interface. J. Phys. Chem. C.

[26] Onizhuk M.O., Panteleimonov A.V., Kholin Y.V., Ivanov V.V. (2018). Dissociation Constants of Silanol Groups of Silic Acids: Quantum Chemical Estimations. J. Struct. Chem..

[27] Contescu C., Popa V.T., Miller J.B., Ko E.I., Schwarz J.A. (1995). Proton Affinity Distributions of TiO2-SiO2 and ZrO2-SiO2 Mixed Oxides and Their Relationship to Catalyst Activities for 1-Butene Isomerization. J. Catal..

[28] Buszewski B., Bocian S., Rychlicki G., Matyska M., Pesek J. (2012). Determination of Accessible Silanols Groups on Silica Gel Surfaces Using Microcalorimetric Measurements. J. Chromatogr. A.

[29] Comas-Vives A. (2016). Amorphous SiO2 Surface Models: Energetics of the Dehydroxylation Process, Strain, Ab Initio Atomistic Thermodynamics and IR Spectroscopic Signatures. Phys. Chem. Chem. Phys..

[30] Fan H.-F., Li F., Zare R.N., Lin K.-C. (2007). Characterization of Two Types of Silanol Groups on Fused-Silica Surfaces Using Evanescent-Wave Cavity Ring-down Spectroscopy. Anal. Chem..

[31] Jain S.K., Tabassum T., Li L., Ren L., Fan W., Tsapatsis M., Caratzoulas S., Han S., Scott S.L. (2021). P-Site Structural Diversity and Evolution in a Zeosil Catalyst. J. Am. Chem. Soc..

[32] Al-Amin K., Kawsar M., Mamun M.T.R.B., Sahadat Hossain M. (2025). Fourier Transform Infrared Spectroscopic Technique for Analysis of Inorganic Materials: A Review. Nanoscale Adv..

[33] Imparato C., Bifulco A., Malucelli G., Aronne A. (2023). Solids Containing Si-O-P Bonds: Is the Hydrolytic Sol-Gel Route a Suitable Synthesis Strategy?. J. Solgel Sci. Technol..

[34] Sułowska J., Wacławska I., Olejniczak Z. (2013). Structural Studies of Copper-Containing Multicomponent Glasses from the SiO2–P2O5–K2O–CaO–MgO System. Vib. Spectrosc..

[35] Saddik R., Hammoudan I., Tighadouini S., Roby O., Radi S., Al-Zaben M.I., Ben Bacha A., Masand V.H., Almarhoon Z.M. (2022). Mesoporous Silica Modified with 2-Phenylimidazo[1,2-a] Pyridine-3-Carbaldehyde as an Effective Adsorbent for Cu(II) from Aqueous Solutions: A Combined Experimental and Theoretical Study. Molecules.

[36] Li C., Zhao J., Zhang Y. (2021). Study on Adsorption Behavior of Nickel Ions Using Silica-Based Sandwich Layered Zirconium-Titanium Phosphate Prepared by Layer-by-Layer Grafting Method. Nanomaterials.

[37] Sarafraz H., Alahyarizadeh G., Minuchehr A., Modaberi H., Naserbegi A. (2019). Economic and Efficient Phosphonic Functional Groups Mesoporous Silica for Uranium Selective Adsorption from Aqueous Solutions. Sci. Rep..

[38] Li D., Wen Y., Hu L., Xu X., Spencer B.S., Egodawatte S., Larsen S.C., Tang Y. (2024). Phosphonate Functionalized Magnetic Mesoporous Silica for Rare Earth Element Recovery from Citrate Assisted Solid Waste Extracts. Appl. Geochem..

[39] Noh H.R., Yoon S.B., Kim T.-H., Lee D.W., Lim S.H., Kim J.-Y. (2025). Pore-Expanded Phosphate-Functionalized Mesoporous Silica Microsphere Composites: One-Pot Synthesis and Application for Removing Uranium from Water. Sep. Purif. Technol..

[40] Khan M., Al- Ghouti M.A., Khraisheh M., Shomar B., Hijji Y., Tong Y., Mansour S., Nasser M.S. (2023). Synthesis of Nanostructured Novel Ion-Imprinted Polymer for Selective Removal of Cu^2+^ and Sr^2+^ Ions from Reverse Osmosis Concentrated Brine. Environ. Res..

