# Supplementary material for: Molecular Imprinting of Phosphate Moieties into the Silica Matrix as a Novel Phosphorus Rechargeable System for Copper Ions Adsorption
Source: Polymers (Basel). 2026 Jul 18;18(14):1759. doi: 10.3390/polym18141759 (PMC13431410; doi:10.3390/polym18141759)
Supplement: Supplementary file 1 [file polymers-18-01759-s001.zip › polymers-4430223-supplementary.pdf]

SUPPLEMENTARY MATERIALS

Molecular Imprinting Cavities of Phosphate Moieties into the Silica Matrix as a Novel Phosphorus Rechargeable System for Copper Ions Adsorption

José A. Gutiérrez-Ortega[1], Jessica Badillo-Camacho[1,2*], Rene G. Moran-Salazar[2], Sergio Gómez-Salazar[3], Ilya G. Shenderovich[4], Yenni G. Velázquez-Galván[5], Ricardo Manríquez-González[1*]

[1]Department of Wood, Cellulose and Paper, University of Guadalajara-CUCEI; Km 15.5 of Carretera Guadalajara-Nogales, Zapopan, Jalisco, Mexico 45020; joseantonio.gutierrez@academicos.udg.mx;

[2]Department of Chemistry, Universidad de Guadalajara-CUCEI, Blvd. Marcelino García Barragán #1421, Guadalajara 44430, Jalisco, Mexico; rene.moran@academicos.udg.mx

[3]Department of Chemical Engineering, Blvd. Marcelino García Barragán # 1421, Guadalajara, Jalisco, Mexico 44430; sergio.gomez@cucei.udg.mx

[4] Faculty of Chemistry and Pharmacy, University of Regensburg, Universitaetstrasse 31, 93053 Regensburg, Germany

[5]Department of Mathematics and Physics, Instituto Tecnológico y de Estudios Superiores de Occidente (ITESO), Periférico Sur Manuel Gómez Morín 8585, Tlaquepaque, Jalisco, México 45604; yenni.velazquez@iteso.mx

*Correspondence: jessica.bcamacho@academicos.udg.mx and ricardo.manriquez@academicos.udg.mx

ORCID: https://orcid.org/0000-0001-5403-1687, https://orcid.org/0000-0002-6899-0149

Table S1. Textural surface parameters of SG-PO adsorbents.

Sample	BET Surface (Area m^2/g)	Mean Pore Volume (cm^3/g)	Pore Size (Å)
SG-P1	498.15	0.1785	42.40
SG-P2	488.56	0.6204	47.80
SG-P3	586.68	0.4726	31.26
SG-P4	515.12	0.1474	36.58
SG-P5	522.61	0.3656	39.22
SG-P6	382.17	0.3856	44.29
SG	849.50	0.4558	36.17

Table S2. Quantitative analysis of the elemental composition by weight of SG-P and SG-P-Cu, collected from different areas by SEM-EDX method.

Sample	Wt%				
	O	Si	C	P	Cu
SG-P	52.3 (3.27) [a]	26.9±0.1 (0.96) [a]	20.0 (1.67) [a]	0.8 (0.026) [a]	N.D
SG-P-Cu	42.6 (2.66) [a]	32.1 (1.14) [a]	24.4 (2.03) [a]	0.7 (0.023) [a]	0.20 (0.003) [a]
SG (P-Cu desorbed)	55.3 (3.46) [a]	31.6 (1.13) [a]	13.0 (1.08) [a]	N.D	N.D
SG-P (H_3PO_4 loaded)	44.87 (2.80) [a]	31.59 (1.13) [a]	22.71 (1.89) [a]	0.53 (0.017) [a]	N.D
SG-P-Cu (H_3PO_4 loaded + Cu)	52.83 (3.3) [a]	38.57 (1.37) [a]	7.42 (0.62) [a]	0.54 (0.017) [a]	0.51 (0.008) [a]
SG (P-Cu desorbed)	49.32 (3.08)[a]	37.99 (1.36)[a]	12.69 (1.06)[a]	N.D	N.D
SG-P (H_3PO_4 loaded)	46.19 (2.89) [a]	36.01 (1.29) [a]	16.37 (1.36) [a]	1.43 (0.046) [a]	N.D
SG-P-Cu (H_3PO_4 loaded + Cu)	46.89 (2.93) [a]	38.78 (1.39) [a]	12.39 (1.03) [a]	1.41 (0.045) [a]	0.53 (0.008) [a]

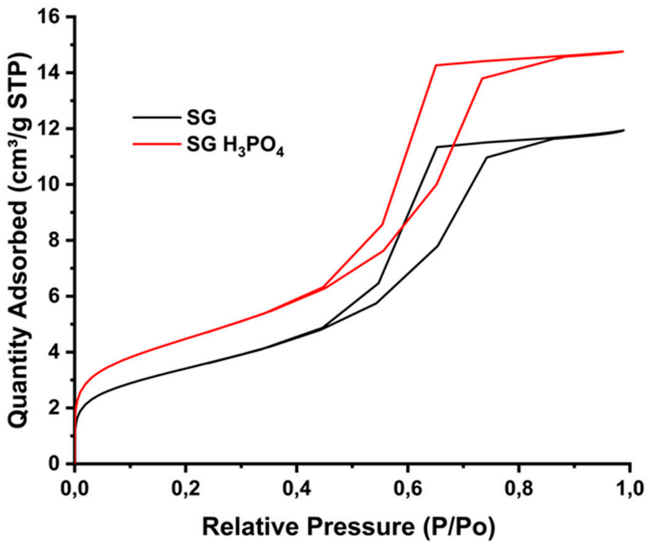

Figure S1. Nitrogen adsorption-desorption isotherms of: SG and SG treated with H_3PO_4.

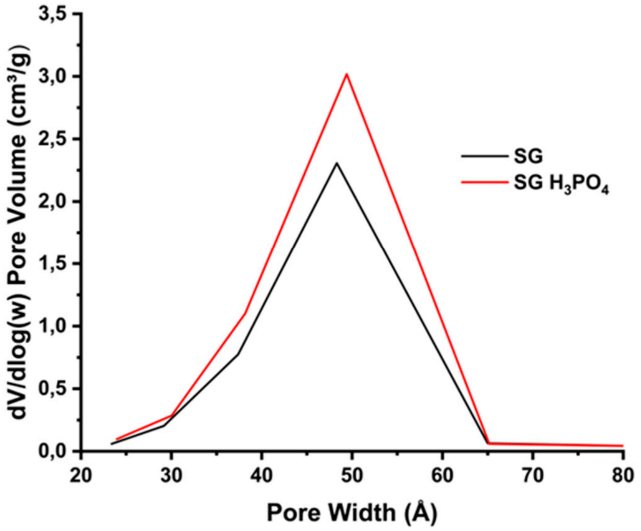

Figure S2. Pore size distribution of: SG and SG treated with H_3PO_4.

Table S3. Textural surface parameters of SG and SG treated with H_3PO_4.

Sample	BET Surface (Area m²/g)	Mean Pore Volume (cm³/g)	Pore Size (Å)
SG	269.19	0.4286	45.25
SG H_3PO_4	354.34	0.5324	44.96

S3. Adsorption kinetics and equilibrium isotherm

S3.1. Adsorption kinetics

S3.1.1 Adsorption kinetic experiments

Adsorption kinetic experiments were carried out using the reference silica (TEOS) and the phosphate-functionalized silica materials SG-P1, SG-P3, and SG-P5. In each experiment, 50 mg of adsorbent were mixed with 2.0 mL of a 106.1 mg L^{-1} Cu(II) solution adjusted to pH 5.0. The suspensions were agitated at 120 rpm in a thermostatically controlled shaker maintained at 303.15 K (30 °C). Independent adsorption experiments were carried out for each contact time (5, 10, 15, 30, 45, 60, 90, 120, and 180 min). At the end of each experiment, the suspension was filtered, and the residual Cu(II) concentration in the filtrate was determined by flame atomic absorption spectrophotometry (Varian model 220). The amount of Cu(II) adsorbed at time t, qt (mg g^{-1}), was calculated from the mass balance according to:

$$q = \frac{V\,(Ci - Ct)}{m} \qquad \text{(S1)}$$

where V is the solution volume (L), Ci and Ct are the initial and residual Cu(II) concentrations (mg L^{-1}), respectively, and m is the mass of adsorbent (g).

The experimental kinetic data were fitted by nonlinear regression using the pseudo-first-order (PFO), pseudo-second-order (PSO), intraparticle diffusion (Weber–Morris), and Avrami models. Parameter estimation was performed with the Levenberg–Marquardt algorithm implemented in OriginPro 2021.

S3.1.2 Discussion

Figure S3 compares the nonlinear fitting of four kinetic models to the experimental Cu(II) adsorption data for the synthesized materials: (a) pseudo-first-order (PFO), (b) pseudo-second-order (PSO), (c) intraparticle diffusion (Weber–Morris), and (d) Avrami model. A silica sample synthesized exclusively from TEOS without the phosphate precursor (TEPO) was included as a reference material to evaluate the contribution of the phosphate functional groups to Cu(II) adsorption. Model selection was based on the coefficient of determination (R^2) and the agreement between the experimental and calculated adsorption capacities. Although the Avrami model (Figure S3d) produced the highest R^2 values for TEOS, SG-P1 and SG-P5, it yielded unrealistic kinetic parameters and poorer agreement between the calculated and experimental adsorption capacities, particularly for SG-P3. In contrast, the pseudo-first-order model (Figure S3a) provided physically meaningful kinetic parameters together with consistently good agreement between the calculated and experimental adsorption capacities for all phosphate-functionalized materials. The intraparticle diffusion model (Figure S3c) showed the poorest overall fitting, indicating that intraparticle diffusion is not the only rate-controlling step during Cu(II) adsorption. The complete fitting parameters are summarized in Table S4.

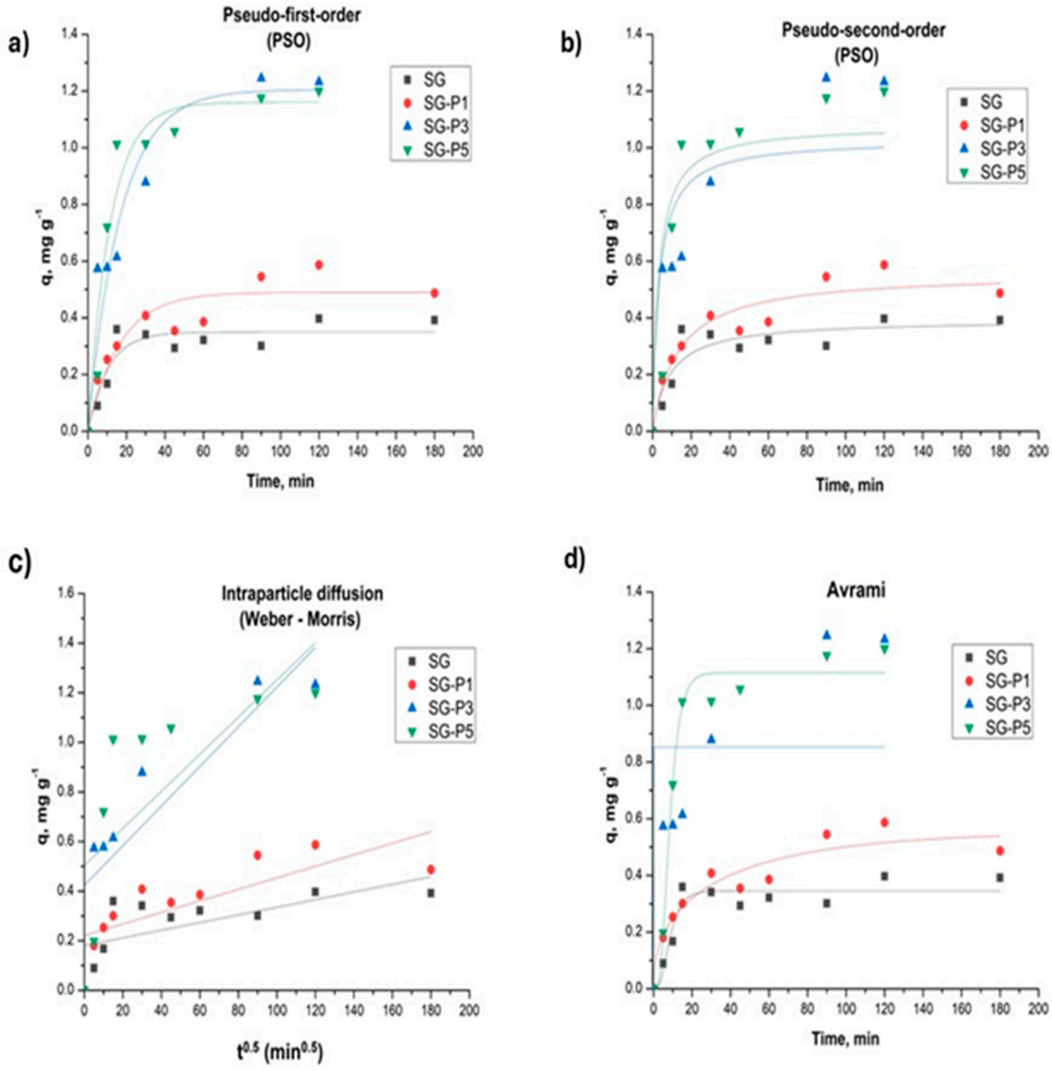

Figure S3. Experimental adsorption kinetics of Cu(II) on phosphate-functionalized silica materials synthesized with different TEOS:TEPO molar ratios and nonlinear fitting to different kinetic models: (a) pseudo-first-order (PFO), (b) pseudo-second-order (PSO), (c) intraparticle diffusion (Weber-Morris), and (d) Avrami.

Table S4. Kinetic parameters obtained by nonlinear regression using the Levenberg–Marquardt algorithm implemented in OriginPro 2021.

Model	Parameter	TEOS	SG-P1	SG-P3	SG-P5
Experimental	$q_{max,exp}, mg\ g^{-1}$	0.40	0.59	1.37	1.44
Pseudo-first-order (PFO)	$q_{max,calc}, mg\ g^{-1}$	0.35 ± 0.02	0.49 ± 0.03	1.20 ± 0.10	1.16 ± 0.07
	k_1, min^{-1}	0.095 ± 0.028	0.060 ± 0.016	0.059 ± 0.014	0.087 ± 0.018
	R^2	0.8620	0.8737	0.9133	0.9410
Pseudo-second-order (PSO)	$q_{max,calc}, mg\ g^{-1}$	0.39 ± 0.04	0.56 ± 0.04	1.03 ± 0.08	1.08 ± 0.08
	$k_2, g\ mg^{-1}\ min^{-1}$	0.39 ± 0.04	0.56 ± 0.04	1.03 ± 0.08	1.08 ± 0.08
	R^2	0.8506	0.9148	0.8134	0.8077
Intraparticle diffusion (Weber–Morris)	$K_{IPD}, mg\ g^{-1}\ min^{-1/2}$	0.0031 ± 0.0012	0.0047 ± 0.0014	0.0160 ± 0.0041	0.0150 ± 0.0060
	C	0.181 ± 0.048	0.221 ± 0.053	0.423 ± 0.120	0.503 ± 0.170
	R^2	0.4482	0.5975	0.7520	0.5104
Avrami	$q_{max,calc}, mg\ g^{-1}$	0.35 ± 0.02	0.55 ± 0.08	0.85 ± 0.15	1.12 ± 0.03
	k_A	0.230 ± 0.119	0.015 ± 0.015	124.76 ± 0.00	0.259 ± 0.058
	n_A	4.36 ± 4.58	0.41 ± 0.16	100.74 ± 0.00	5.44 ± 2.68
	R^2	0.8987	0.9277	0.5504	0.9835

S3.2. Equilibrium isotherm

S3.2.1 Adsorption equilibrium isotherm experiment

Adsorption equilibrium experiments were carried out using the selected phosphate-functionalized silica (SG-P5). In each experiment, 50 mg of adsorbent were mixed with 2.0 mL of Cu(II) solutions with initial concentrations ranging from 10 to 1000 mg L^{-1}. The solution pH was adjusted to 5.0, and the suspensions were agitated at 120 rpm for 3 h in a thermostatically controlled shaker maintained at 303.15 K (30 °C) to ensure equilibrium.At the end of the adsorption period, the suspensions were filtered, and the equilibrium Cu(II) concentration was determined by flame atomic absorption spectrophotometry (Manufacturer and model). The equilibrium adsorption capacity, q (mg g^{-1}), was calculated by mass balance using Eq. (S1) with Ct = Ce where Ce is the Cu equilibrium concentration. The equilibrium data were fitted by nonlinear regression using the Langmuir, Freundlich, Tóth, Sips (Langmuir–Freundlich), Temkin, and S-shape isotherm models. Parameter

estimation was performed using the Levenberg–Marquardt algorithm implemented in OriginPro 2021.

S3.2.2 Discussion

Figure S4 compares the nonlinear fitting of different adsorption isotherm models to the experimental equilibrium data: Langmuir (Figure S4a), Freundlich (Figure S4b), Tóth (Figure S4c), Sips (Langmuir–Freundlich) (Figure S4d), Temkin (Figure S4e), and S-shape (Figure S4f). Model selection was based on the coefficient of determination (R^2), the agreement between the experimental and calculated adsorption capacities ($q_{max\ calc}$), and the overall quality of the nonlinear regression. Among the evaluated models, the Tóth isotherm (Figure S4c) provided the best overall fit, yielding the highest R^2 value together with the closest agreement between the experimental ($q_{max\ exp}$=2.09 mg g^{-1}) and calculated ($q_{max\ calc}$=2.06 mg g^{-1}) adsorption capacities. This behavior is consistent with the structural heterogeneity expected for phosphate-functionalized silica prepared by the sol-gel method. The complete fitting parameters for all adsorption isotherm models are summarized in Table S5.

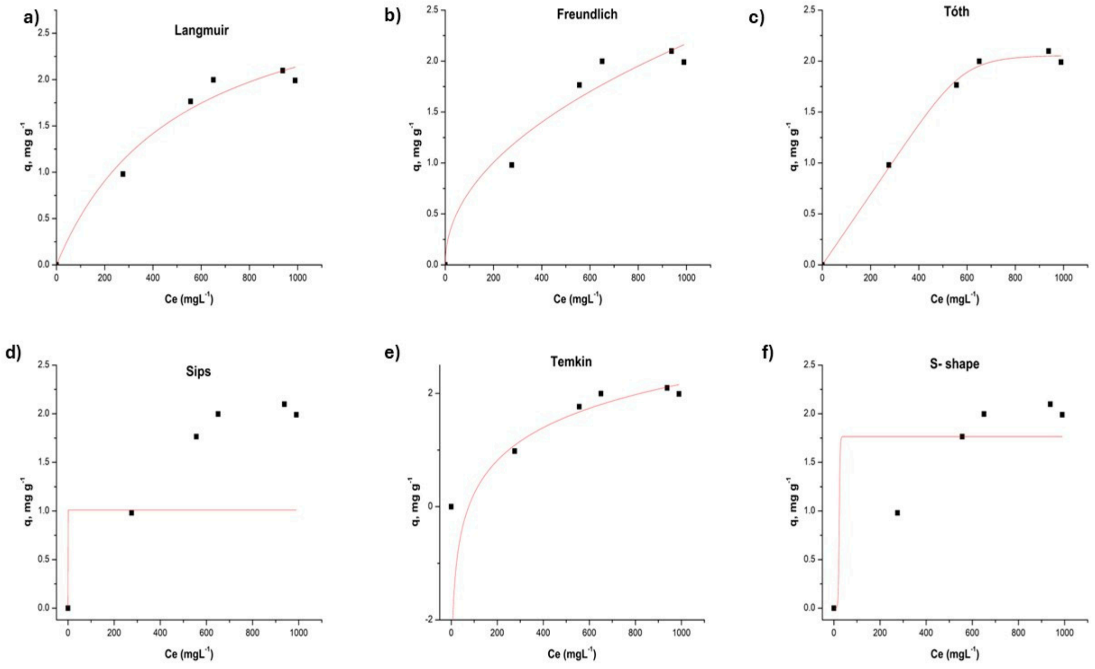

Figure S4. Experimental Cu(II) adsorption isotherm for the selected phosphate-functionalized silica together with nonlinear fitting using different adsorption isotherm models: (a) Langmuir, (b) Freundlich, (c) Tóth, (d) Sips (Langmuir–Freundlich), (e) Temkin, and (f) S-shape model

Table S5. Isotherm parameters obtained by nonlinear regression using the Levenberg–Marquardt algorithm implemented in OriginPro 2021.

Model	Parameter	SG-P5
Experimental	$q_{max,exp}, mg\ g^{-1}$	2.09
Langmuir	$q_{max,calc}, mg\ g^{-1}$	3.25 ± 0.56
	$K_{L}, L\ mg^{-1}$	0.00193 ± 0.00079
	R^2	0.9753
Freundlich	K_F	0.0795 ± 0.0647
	n	2.089 ± 0.544
	R^2	0.9608
Tóth	$q_{max,calc}, mg\ g^{-1}$	2.06 ± 0.05
	b (L mg^{-1})	0.00168 ± 0.00010
	t	7.733 ± 3.925
	R^2	0.9968
Sips (Langmuir–Freundlich)	$q_{max,calc}, mg\ g^{-1}$	1.01 ± 0.50
	K_{FL}	99.61 ± 0.00
	n	0.104 ± 0.00
	R^2	-0.0789
Temkin	A_T	0.0132 ± 0.0048
	b_T	2952.59 ± 498.47
	R^2	0.9754
S-shape	N_0	1.77 ± 0.24
	k_1	0.500 ± 0.00
	k_2	$1.02 \times 10^5 \pm 3.11 \times 10^9$
	R^2	0.7576
